# Application of mosquito repellent coils and associated self-reported health issues in Ghana

**DOI:** 10.1186/s12936-016-1126-8

**Published:** 2016-02-04

**Authors:** Jonathan N. Hogarh, Philip Antwi-Agyei, Kwasi Obiri-Danso

**Affiliations:** Department of Environmental Science, College of Science, Kwame Nkrumah University of Science and Technology (KNUST), Kumasi, Ghana

**Keywords:** Malaria, Mosquito coil, Mosquito net, Indoor residual spraying, Acute respiratory infections (ARI), Indoor air pollution

## Abstract

**Background:**

The use of mosquito coils has gained widespread patronage in malaria-endemic countries, even though it is not a recommended preventive measure for avoiding mosquitoes. Mosquito coils contain insecticides, which are expected to vaporize slowly once the coil is lit, to provide protection against the mosquito. The mosquito coil base material contains a variety of compounds capable of burning slowly to gradually release the insecticide. The mosquito coil smoke, however, is potentially a source of indoor air pollution with implications for acute respiratory infections (ARI) and other illnesses. The present study investigated the application of mosquito coils and associated self-reported health issues in Ghana.

**Methods:**

A cross-sectional study was undertaken in which questionnaires were randomly administered to 480 households across four districts in Ghana. Respondents who exclusively applied mosquito coils were grouped as test cohort, while those who did not apply any mosquito repellency method constituted a control cohort.

**Results:**

The test group that applied mosquito coils reported malaria incidence rate of 86.3 %. The control group that did not apply any mosquito repellency method reported an incidence rate of malaria at 72.4 %. Chi square analysis suggested that the observed difference was statistically significant (*x*^*2*^ = 4.25; p = 0.04). The number of respondents who reported symptoms of cough from mosquito coil application (52.6 % incidence rate) was marginally greater than their counterparts who did not apply coils (46.1 % incidence rate). It was also found that respondents with shortage of breath, which was used as a proxy for ARI, were more likely to have applied mosquito coil.

**Conclusions:**

The application of mosquito coils did not necessarily reduce the incidence of malaria in the study communities. It however presented a potential respiratory risk factor, which should be further investigated by critically examining exposure to particulate matter emissions from burning coils.

## Background

The World Health Organization (WHO) estimates that there were approximately 207 million cases of malaria worldwide in 2013, with an estimated 627,000 deaths [1]. The worst affected countries were in Africa and the worst affected cohort were children aged under 5 years. A key aspect of malaria control programmes, globally, is to control the vector that causes malaria—the mosquito. WHO recommended practices, in this regard, include the use of insecticide-treated nets (ITN) and indoor residual spraying (IRS). In Ghana, the goal is to achieve 100 % coverage of households that own ITNs, with at least 80 % of the general populace sleeping under ITNs [2]. However, only about 50 % of households apparently own ITNs in the country, of which the estimated usage rate is 57 % [3]. These estimates suggest that only about a quarter of the population in Ghana (25 %) is actually using mosquito nets to control the vector. Relatively few households can afford insecticide-based IRS because of the cost involved [4]. A greater majority of households, especially the urban poor and rural dwellers, use mosquito coils to control/repel the mosquito. The consumer market for mosquito coils was estimated at one billion dollars globally in 2006, accounting for almost 12 % of the global market for pesticides in that year [5]. Currently, the use of mosquito coils occupies a certain niche in the control of mosquitoes in poor countries that need to be adequately researched.

Mosquito coils are not officially included in malaria control programmes in Ghana, yet they are widely patronized in the country, especially among the rural and urban poor. The patronage of the mosquito coil extends across tropical Africa, Asia and South America and it is used to control mosquitoes especially at household level [6–9]. One reason why the mosquito coil is so highly patronized in developing countries is because it is cheap and accessible to the poor [10].

Despite its potential benefit as a mosquito repellent, the mosquito coil may generate undesirable emissions, which constitute a potential source of indoor air pollution [7]. The base material of the mosquito coil is mainly organic in nature, consisting of organic fillers, binders, dyes, and other additives capable of burning slowly to gradually release the insecticide with smoke. Mosquito coil smoke emissions may contain pollutants such as carbon monoxide, particulate matter, polycyclic aromatic hydrocarbons (PAHs), aldehydes, ketones and a suite of volatile organic compounds (VOCs) [7]. These are mostly products of incomplete combustion, a reflection of the fact that most mosquito coils are designed to burn inefficiently to facilitate the slow release of the insecticide. Exposures to these airborne emissions have various health implications. For instance, particulate matter may trigger acute respiratory infections (ARIs), while VOCs and PAHs are potentially carcinogenic [11, 12]. The aim of this study was to investigate the rate of application of mosquito coils as compared with recommended mosquito avoidance methods in Ghana and potential health effects that may be associated with the application of these coils.

## Methods

### Study area

**Fig. 1:**
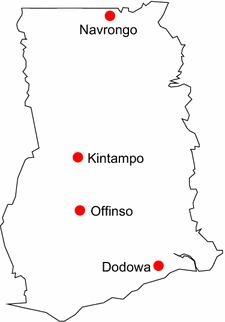
Study sites where questionnaires were administered in Ghana

The study was undertaken in four districts in Ghana: Dodowa (in southern Ghana), Kintampo and Offinso (in mid Ghana) and Navrongo (in northern Ghana). These districts were selected because they are among malaria-endemic districts in the country [13, 14], as well as belong to different ecological zones (Fig. 1).

### Questionnaire administration

A cross-sectional study was undertaken in which questionnaires were randomly administered to households in the four selected districts in June 2015. Respondents were adequately briefed about the project and their consents were appropriately sought before administration of the questionnaires. In all, 480 household questionnaires were administered, with 120 questionnaires administered in each of the four districts. The questionnaire survey was intended to, first of all, provide information on the rate of application of mosquito coils as compared with other mosquito avoidance methods. Secondly, the survey was intended to provide a randomized controlled analysis between mosquito coil-using test group and non-coil-using control group. Therefore, respondents who exclusively applied mosquito coils were eventually grouped as test cohort (with 95 members), while those who did not apply any mosquito repellency method constituted a control cohort (with 76 members). The head of the household (or the representative) was interviewed and each interview lasted between 30 and 45 min.

### Statistical analysis

Responses captured by questionnaires were coded and organized in Statistical Package for Social Sciences (SPSS). The test and control groups were subjected to Chi square analysis and differences amounting to p-values less than 0.05 were considered significant. Multiple regression analysis was used to evaluate relevant predictor factors underpinning the dataset.

### Ethical clearance

Ethical clearance for the study was obtained from the Committee on Human Research Publication and Ethics (CHRPE), Kwame Nkrumah University of Science and Technology in Ghana, as well as from the WHO Ethics Committee.

## Results

### Application of mosquito repellency methods

**Fig. 2:**
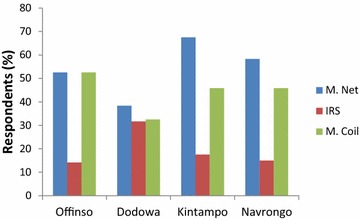
Percentage respondents who indicated a current application of the mosquito avoidance methods. *M Net* mosquito net; *IRS* insecticide residual spray; *M Coil* mosquito coil

Respondents’ preferences for three different mosquito avoidance methods, i.e., application of mosquito net, IRS and mosquito coils, were investigated. Figure 2 shows the current applications of these methods in the respective districts. The average patronage of mosquito avoidance methods among the districts was as follows: mosquito nets 54.2 %, IRS 19.6 % and mosquito coils 44.2 %. The cumulative average patronage was greater than 100 % because some respondents reported co-application of different mosquito avoidance methods, that is, they applied more than one method. The 54 % of respondents using mosquito nets fell far below the Ghana Health Service (GHS) target of 80 % [2]. The value was however consistent with the 57 % usage rate reported by Adjei and Gyimah [3]. The difference between the number of respondents who applied mosquito net and those who applied mosquito coil was not statistically significant at 95 % confidence level (p = 0.06). It presupposed that as many respondents applied mosquito coils as patronage for mosquito nets. The high patronage of the mosquito coil partly contributed to the difficulty in achieving high rate of patronage for mosquito net application in the study districts, despite the free provision of these nets to households [9]. The use of IRS was generally minimal due to its relative high cost (Fig. 2). The Dodowa district, located near the capital city, Accra, recorded relatively increased application of IRS in comparison to the other districts. Consequently, the applications of mosquito net and coil were relatively reduced at Dodowa.

**Fig. 3:**
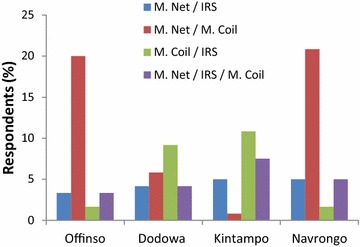
Co-application of mosquito avoidance methods in Ghana. *M Net* mosquito net; *IRS* insecticide residual spray; *M Coil* mosquito coil

With regard to the co-applications, those that applied a combination of mosquito net and coil were in the majority at Offinso and Navrongo districts (Fig. 3). There was however a sharp contrast in co-application of mosquito net and coil at Kintampo. The reason for this difference was not clear. At Dodowa and Kintampo, co-application of mosquito coil and IRS was comparatively high. On average, 11.9 % of respondents co-applied mosquito net and coil, 5.8 % co-applied mosquito coil and IRS, 4.4 % co-applied mosquito net and IRS, and 5.0 % co-applied all three methods. Although some respondents had preference for mosquito net, they indicated discomfort in sleeping in the net at certain times, especially in hot weather, such that they sometimes switched to the use of mosquito coils. Hence, the relatively high co-application of mosquito net and coil. A hot sleeping environment under mosquito net has been reported among the most common reasons for non-use of mosquito nets in Ghana [15].

**Fig. 4:**
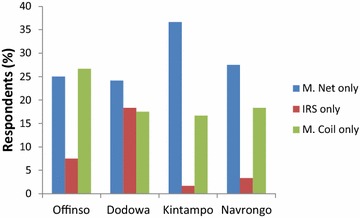
Percentage respondents with exclusive applications of mosquito repellency methods. *M Net* mosquito net; *IRS* insecticide residual spray; *M Coil* mosquito coil

The percentage of respondents with an exclusive application regarding each of the mosquito repellency methods is indicated in Fig. 4. Exclusive application of mosquito net was highest at Kintampo (≈37 %). At an average, 28 % of respondents used only mosquito net, 20 % used only mosquito coil and 7.7 % used only IRS. Respondents at Kintampo indicated that they were provided with free mosquito nets just about a fortnight before the questionnaire administration. This probably influenced the usage rate of the nets in the community. Thus, regular campaigns to provide free mosquito nets to households are desirable to encourage usage of these nets. Contrary views though exist that issuing mosquito nets for free may hinder ownership and usage [9, 16]. This is premised on the fact that people purchase items that they really need and for that reason are inclined to use what was purchased. It is also valid that those who cannot afford a net should be supplied free of charge to encourage usage.

### Distribution of participants over mosquito repellency methods

**Fig. 5:**
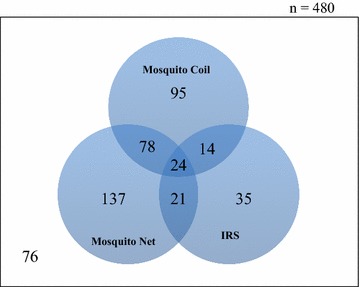
Distribution of respondents based on their application of mosquito repellency methods

The rates of application of mosquito repellency methods from the study districts are summarized in Fig. 5. A total of 260 per 480 respondents applied mosquito net, 211 per 480 applied mosquito coil and 94 per 480 applied IRS. Seventy-six respondents did not apply any mosquito repellency method at all. A high number of co-applications of the mosquito repellency methods existed (137 per 480 respondents). As respondents with co-applications may confound the test-control analysis, they were excluded in the respective cohorts. Therefore, only respondents with exclusive application of mosquito coil were included as test cohort. From Fig. 5, 95 respondents qualified as members of the test group for mosquito coil application. The control cohort was made up of the 76 respondents who did not use any mosquito repellency method at all.

### Comparison between test and control groups

**Fig. 6:**
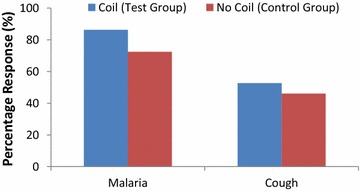
Self-reported episodes of malaria and cough from the test and control cohorts

The test group that applied mosquito coil reported malaria incidence rate of 86.3 % (Fig. 6). The control group that did not apply any mosquito repellency method reported a reduced incidence rate of malaria at 72.4 %. Chi square analysis suggested that the observed difference was statistically significant (*x*^*2*^ = 4.25; p = 0.04). This meant that the application of mosquito coil did not necessarily reduce the incidence rate of malaria in the study communities. It is unclear if this was related to the efficacy of the mosquito coil, or perhaps those who did not apply any of the mosquito repellency methods (the control group) were more cautious in not exposing themselves to mosquito bites at night. It is nevertheless likely that mosquito coils may not be delivering the needed protection against malaria. Hence, their exclusive application has not been recommended [9]. Respondents who reported symptoms of cough from mosquito coil application (52.6 % incidence rate) were marginally greater than their counterparts who did not apply the coil or any other mosquito repellency method (46.1 % incidence rate). The difference between these two groups was not statistically significant (*x*^*2*^ = 0.52; p = 0.47), even though earlier studies suggested a marginally significant positive association of cough with mosquito coil use [17].

### Multiple regression analysis

**Fig. 7:**
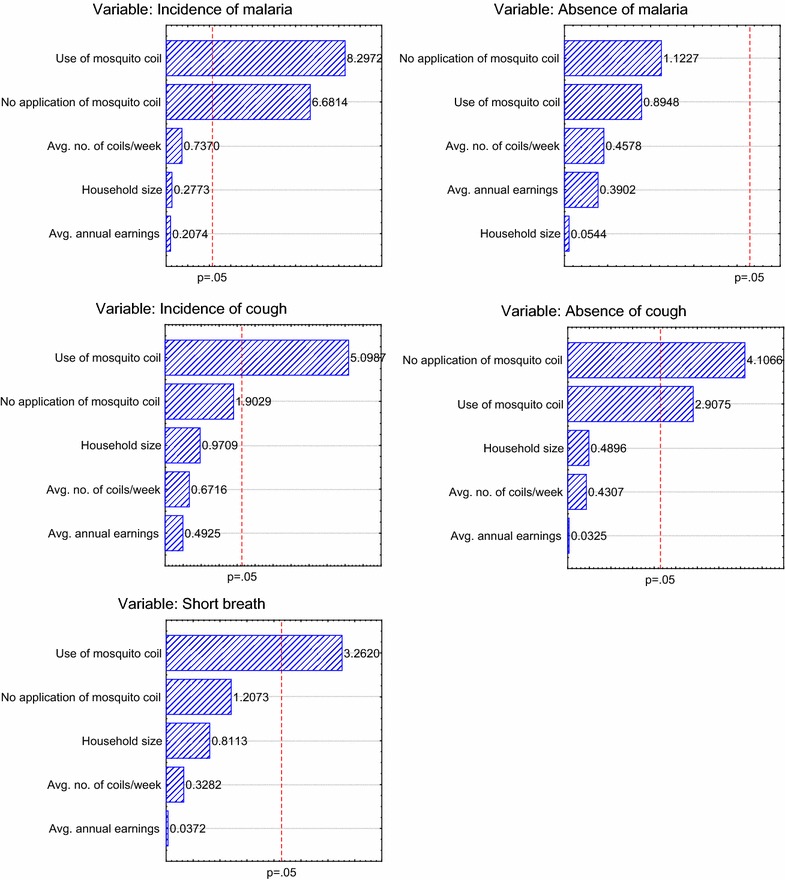
*Pareto chart* of t-values for coefficients from multiple regression analysis

The data were further segregated based on household size (Table 1) and subjected to multiple regression analysis. Household size, annual average income, the use or non-use of mosquito coil, and the average number of mosquito coils applied in households were used as the independent or predictive variables, with frequency of incidence or absence of malaria, cough and short breath as the dependent variables. The output of this analysis is displayed in the form of Pareto chart of t-values for the regression coefficients (Fig. 7). The use and non-use of mosquito coil were significant as predictive variables of the frequency of malaria incidence in households (p < 0.0001) (Fig. 7a). Thus, respondents who used mosquito coil or did not use mosquito coil were both likely to have malaria. None of the independent variables related significantly to the absence of malaria in households (p > 0.2 in each case) (Fig. 7b), which suggested that the absence of malaria was not dependent on any of the independent factors investigated. The effect size (regression coefficient) regarding the application of mosquito coil was significant whether respondents reported issues of cough or not (Fig. 7c, d). This may explain why the test and control groups did not differ with respect to the potential issue of cough (Fig. 6). Thus, the use of mosquito coil was not necessarily a predictor variable of cough in the households, although those who did not use the coil appeared less likely to suffer from cough (Fig. 7d). Respondents with short breath, which was used as a proxy for ARI, were more likely to have applied mosquito coil (Fig. 7e). These findings are consistent with earlier suggestions that mosquito coil burning is a respiratory risk factor, although did not prove causality [17].

**Table 1:** Participants’ responses segregated by household size

Household size	Frequency of households	Average annual earnings (GHC)	Frequency of respondents who reported malaria	Frequency of respondents without malaria	Frequency of respondents who used mosquito coil	Frequency of respondents who did not use any preventive method	Average no. of coils/week	Frequency of respondents who reported cough	Frequency of respondents without cough	Frequency of respondents with issues of short breath
1	3	667	2	1	2	1	7.0	0	0	0
2	13	654	12	2	5	8	6.8	5	7	5
3	18	688	18	1	9	9	6.6	11	7	5
4	22	675	20	2	14	8	5.4	13	9	7
5	21	625	16	6	12	9	5.9	11	10	5
6	26	841	23	3	16	10	6.0	13	13	10
7	19	694	17	3	10	9	4.4	8	11	4
8	11	1650	10	2	5	6	7.4	5	6	2
9	6	500	6	1	6	0	7.0	3	3	1
10	12	1375	11	1	6	6	6.7	3	9	1
11	4	667	3	1	4	0	12.0	4	0	3
12	3	500	3	0	2	1	8.5	3	0	1
13	1	1000	0	1	0	1	0.0	0	1	0
14	1	500	1	0	0	1	0.0	0	1	0
15	2	5250	2	0	1	1	7.0	1	0	1
16	1	500	1	0	1	0	7.0	1	0	0
17	1	500	0	1	0	1	0.0	0	1	0
20	3	833	1	2	0	3	0.0	2	1	1
21	2	750	1	1	1	1	4.0	0	2	0
24	2	500	2	0	1	1	1.0	2	0	2

## Conclusions

The rate of application of mosquito coil was 44, 10 % lower than the rate of mosquito net application. The usage rate of the coil was nevertheless quite high considering that it is not a recommended method for avoiding the mosquito. The application of mosquito coil did not necessarily reduce the incidence rate of malaria. Respondents who used mosquito coil or otherwise were both likely to have malaria, which portended limited protection from these coils. The use of mosquito coils was associated with self-reported incidence of ARI. It was therefore concluded that the use of mosquito coil may present a respiratory risk factor; this needs to be investigated further by critically examining exposure to particulate matter emissions from burning coils.
